# Experience with onabotulinumtoxinA (BOTOX) in chronic refractory migraine: focus on severe attacks

**DOI:** 10.1007/s10194-011-0294-8

**Published:** 2011-02-05

**Authors:** A. Oterino, C. Ramón, J. Pascual

**Affiliations:** 1 Service of Neurology, University Hospital Marqués de Valdecilla, Santander, Spain; 2 Neuroscience Area, Service of Neurology, University Hospital Central de Asturias, Oviedo, Spain

**Keywords:** Chronic migraine, Chronic refractory migraine, OnabotulinumtoxinA

## Abstract

The objective of this study is to analyse our experience in the treatment of refractory chronic migraine (CM) with onabotulinumtoxinA (BTA) and specifically in its effects over disabling attacks. Patients with CM and inadequate response or intolerance to oral preventatives were treated with pericranial injections of 100 U of TBA every 3 months. The dose was increased up to 200 U in case of no response. The patients kept a headache diary. In addition, we specifically asked on the effect of BTA on the frequency of disabling attacks, consumption of triptans and visits to Emergency for the treatment of severe attacks. This series comprises a total of 35 patients (3 males), aged 24–68 years. All except three met IHS criteria for analgesic overuse. The number of sessions with BTA ranged from 2 to 15 (median 4) and nine (26%) responded (reduction of >50% in headache days). However, the frequency of severe attacks was reduced to an average of 46%. Oral triptan consumption (29 patients) was reduced by 50% (from an average of 22 to 11 tablets/month). Those six patients who used subcutaneous sumatriptan reduced its consumption to a mean of 69% (from 4.5 to 1.5 injections per month). Emergency visits went from an average of 3 to 0.4 per trimester (−83%). Six patients complained of mild adverse events, transient local cervical pain being the most common. Although our data must be taken with caution as this is an open trial, in clinical practice treatment of refractory CM with BTA reduces the frequency of disabling attacks, the consumption of triptans and the need of visits to Emergency, which makes this treatment a profitable option both clinically and pharmacoeconomically.

## Introduction

Chronic migraine, understood as the presence of headache during 15 or more days in a month in patients with migraine history, is an important health problem [1]. It is estimated that about 2% of general population meets the criteria for chronic migraine with or without analgesic overuse [2, 3]. Prevalence in women around 40–50 years of age reaches 5%, thus justifying that almost 5% of consultations to Neurology Services in Spain are caused by this condition [4].

Chronic migraine is important not only on account of its great frequency, but also because it significantly reduces the quality of life of the patients affected and it determines an unquestionable morbidity [5]. On one hand, these patients often develop complications connected with analgesic consumption, such as upper digestive haemorrhage or analgesic nephropathy [6]. On the other hand, they usually associate chronic depression, partly due to the increased frequency of disabling pain [3, 5]. Lastly, recent data suggest the increased frequency of severe attacks related to chronic migraine is a stroke risk factor [7].

Migraine treatment is a complex issue. If analgesic overuse is present, especially opiates or ergotics, withdrawal becomes compulsory. Most patients are in need of a preventive treatment. Even though with the exception of topiramate [8, 9], there are no controlled trials in chronic migraine for the majority of the preventative treatments used in episodic migraine, clinical practice suggests that beta-blockers, amitryptiline, flunarizine and some neuromodulators can be useful when treating these patients. However, a relevant subgroup of these patients does not respond effectively to any of the preventative treatments. These patients met the criteria for chronic refractory migraine [10]. Aggressive options such as bilateral suboccipital stimulation have been tried lately in these patients with poor results. Recently, effectiveness of onabotulinumtoxinA (BTA), BOTOX to be precise, has been proven for preventive treatment of patients with chronic migraine [11–13]. Our goal was to assess our experience in treating those patients with chronic refractory migraine with BTA in daily clinical practice, focusing on its effect on disabling attacks and taking into account various parameters that had not been specifically studied in clinical trials.

## Patients and methods

Subjects involved in this study were patients from our refractory headache clinic and had to satisfy the following requirements: (1) meet the criteria for chronic migraine with or without analgesic overuse; (2) show insufficient response (over a minimum of 6 weeks) or absence of tolerability of beta-blockers, flunarizine, topiramate, valproic acid and amitryptiline; and (3) give their informed consent to pericranial treatment with BTA. All patients with criteria for analgesic abuse had failed in at least one withdrawal attempt. Both patients with fibromyalgia and active depression as well as those overusing ergotics or opiates were excluded. Patients were allowed to continue with preventative oral medication during the treatment with BTA.

Patients were treated with BTA every 3 months. All patients received a minimum of two treatments. The first of them consisted of injecting 100 U into 20 sites (5 units per site) distributed among the muscles of each hemicranium as follows: one site into corrugator, two into frontalis, three into temporalis, two into suboccipitalis, one into semispinalis and one into splenius. In case of insufficient response, the dose was increased up to a maximum of 200 U and 40 sites in accordance with the protocol for phase III trials.

Patients kept a conventional headache diary regularly. Under this study we took into consideration the diary noted in the second month of the last quarter of the treatment and compared it to the one written during the pretreatment. Consequently, we compiled specific information about the effect of BTA on disabling attacks and on consumption of triptans as well as about the visits to Emergency, whether it be a health centre or the hospital, for parenteral treatment of attacks refractory to domiciliary management.

## Results

Our series covers 35 patients (3 males) aged between 24 and 68. Only three of them did not meet the criteria for analgesic overuse. The average of headache days per month before treatment with BTA was 24.7, while that of days with severe, disabling headaches was 8.2 per month. A total of 29 patients were taking oral triptans regularly (an average of 22 pills/month before the study) and 6 were also using subcutaneous sumatriptan (an average of 4.5 injections/month before the study). Only one patient was not undergoing oral preventative treatment. At the time of the evaluation 16 patients were taking one oral preventative, 15 were taking two and 3 patients were undergoing medical tritherapy. Drugs used were in the order: topiramate, 15 patients; amitriptyline, 12; zonisamide, 7; beta-blockers, 5; valproic acid, 4; candesartan 4 and flunarizine, 4.

**Fig. 1:**
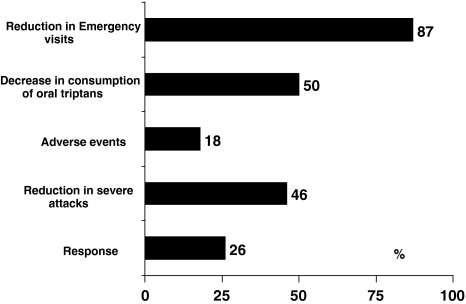
Summary of the main results of this study

The number of treatments ranged between a minimum of 2 and a maximum of 16 (median 4). “Response” (reduction of headache days per month at least by 50%) was observed in 9 patients (26%) and “excellent response” (>75% reduction) in only 2 of them (6%). The average number of days with severe, disabling headache after treatment was 3.8 per month (mean reduction 46%, limits 0–905). Consumption of oral triptans in 29 patients who took them regularly halved (from 22 to 11 oral triptans/month). The 6 patients using subcutaneous sumatriptan went from 4.5 injections/month to 1.5 injections/month (69% reduction). Finally, the average number of visits to casualty department for parenteral treatment went from 3 in the pretreatment quarter to 0.4 (87% reduction) (Fig. 1).

Only six patients (18%) experienced adverse effects, always mildly and temporarily, consisting of cervical pain in four cases and eyebrow asymmetry in two of them.

## Discussion

Our experience in clinical practice conditions brings effectiveness and excellent tolerability to pericranial injections of BTA for the treatment of chronic refractory migraine along the lines of published clinical trials [11–13]. Like in those trials, “response” rate, i.e., the reduction of headache days at least by 50%, was not outstanding. Only one-fourth of the patients met the criteria of response laid down by the International Headache Society and less than 10% fulfilled the criteria for excellent response [14]. If we analyze, however, what occurred in disabling attacks, BTA’s effectiveness was very clear: their frequency halved and the number of visits to Emergency was reduced by almost 90%. These data explain what seems a discrepancy in the results (in terms of response) of the clinical trials, which are not spectacular over placebo, and what happens in clinical practice, where the patient declares to feel clearly better although the number of headache days in the diary has not dropped dramatically. However, even taking into consideration that during our series patients had been refractory to various treatments, we cannot rule out a placebo effect; these data suggest that the effect of BTA in chronic migraine lies in a downward modulation of severe pain attacks, which would then be less invalidating and easier to deal with. This is endorsed by two of our results: both the halved consumption of triptans, drugs these patients only take for their most disabling attacks, and the dramatic decrease in the number of visits to Emergency for parenteral treatment. Again, however, we would like to emphasize that these data must be taken with caution as no placebo arm was included in this trial. This is not a formal pharmacoeconomic study, but its results can help to illustrate the potential cost advantages of this new treatment approach. Consider the following examples: an easy calculation taking into consideration the local average price of oral triptans indicates that the savings—only in oral triptans—per patient and month would be of €101. Savings in casualty department visits, taking into account the official price in our country of €138 per visit without further studies, is even higher.

Our treatment protocol differs in some aspects from the one carried out in phase III of the clinical trials. In this study we administered an initial dose of 100 U and we only raised it to 150–200 U in those patients whose response was insufficient. The same happened with the number of points injected, which was lower (20 compared to a minimum of 31). Same muscular groups were injected, except for the trapezius, which was left with no treatment. Positive open results for BTA with the same dose and lower than ours exist [15–17]. Therefore, these differences are logical since the protocol of the clinical trial tries to guarantee that a potential lack of effectiveness is not due to an insufficient dose, as against the daily clinical practice that tries to optimize the dose and comfort of the patient.

To conclude, we would like to highlight that BTA has been useful in our experience with patients with chronic refractory migraine who showed analgesic overuse in most cases. Although we excluded patients abusing of ergotics and opiates from the study, these results indicate, along the lines of phase II results [18–20], that patients with chronic migraine and analgesic overuse can improve specifically with preventative treatment, in this instance BTA.
